# Variability in Fish Environmental DNA Concentration in Coastal Ecosystems at Hierarchical Levels: Focusing on the Magnitude, Structure, and Environmental Dependence

**DOI:** 10.1002/ece3.74002

**Published:** 2026-07-12

**Authors:** Toshiaki S. Jo, Hiroaki Murakami

**Affiliations:** ^1^ Graduate School of Informatics Kyoto University Kyoto Kyoto Japan; ^2^ Maizuru Fisheries Research Station, Field Science Education and Research Center Kyoto University Maizuru Kyoto Japan; ^3^ Graduate School of Agricultural Science Tohoku University Sendai Miyagi Japan

**Keywords:** environmental DNA (eDNA), fisheries management, quantitative real‐time PCR (qPCR), Taylor's power law (TPL), *Trachurus japonicus*, variance

## Abstract

Understanding variability in environmental DNA (eDNA) concentration is essential for improving the precision of quantitative eDNA analyses. While previous laboratory and field studies suggest that technical variation arising from sampling and PCR processes is relatively small, the hierarchical structure of this variability and its environmental dependence remain poorly understood. In this study, we conducted spatiotemporally replicated seawater sampling from a coastal ecosystem, quantified Japanese jack mackerel ( *Trachurus japonicus* ) eDNA concentrations using quantitative real‐time PCR (qPCR), and assessed the magnitude, structure, and environmental drivers of eDNA variability across multiple levels. Variance component analysis revealed that more than 90% of the total variance in eDNA concentration was explained by differences among sampling sites and time points, reflecting differences in organismal distribution and dynamics, whereas sampling and PCR steps together contributed less than 10%. Using Taylor's Power Law, we demonstrated that the relationship between mean eDNA concentration and variance differed across hierarchical levels, with stronger mean–variance scaling at larger spatial and temporal scales. Notably, the relative contribution of the PCR level to the total variance increased substantially under low‐concentration conditions, indicating that stochastic measurement error becomes dominant when eDNA is scarce. We also demonstrated that the PCR‐level variability (Coefficient of variance; CV) increased with chlorophyll‐α and decreased with pH, whereas no significant environmental effects were detected at the sample level. These results suggest that different mechanisms govern variability at each hierarchical level, including ecological processes, physical heterogeneity, and biochemical constraints. Our findings highlight that optimal sampling and replication strategies should be tailored to expected eDNA concentrations and environmental conditions in the field, helping provide a framework for maximizing signal‐to‐noise ratios in quantitative eDNA studies.

## Graphical abstract

Differences in eDNA concentration heterogeneity under the TPL framework suggest a concentration‐dependent survey design to minimize noise in eDNA quantification. In addition, whilst spatial and temporal variations in eDNA concentration could be primarily determined by ecological factors such as the distribution and dynamics of individuals, variations between samples may rely on physical and hydrological factors (transport, water movement, and fine‐scale heterogeneity), and variations between PCR replicates may rely on biochemical factors (water chemistry, compounds, and environmental gradient), respectively. Increasing the effort devoted to technical steps that are sensitive to specific environmental conditions and ecosystems may be an effective way to reduce quantification noise.

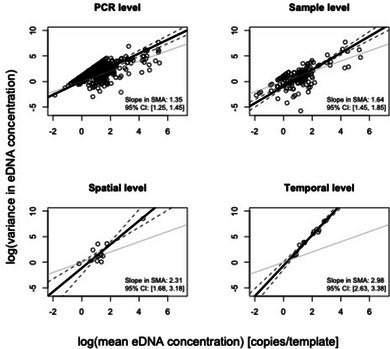

## 1 Introduction

Prompt and precise monitoring of species distribution and abundance is pivotal for effective conservation management and fisheries resource assessment (Matthews and Whittaker 2015; Hansen et al. 2018). For this purpose, visual observation and capture‐based surveys (e.g., angling, electrofishing, and trawling) have traditionally been conducted. However, these methods also have some technical limitations, including damaging the species and their habitat, time‐ and labor‐intensiveness, and/or a lack of taxonomic resolution especially for cryptic species and egg and larval stages (Darling and Mahon 2011; Evans and Lamberti 2018). Against them, environmental DNA (eDNA; extra‐organismal DNA released as the epidermis, mucus, feces, and other tissue fragments into the environment) analysis has the potential to obtain a ‘snapshot’ of species distribution quickly at a broad scale. In the analysis, eDNA in a water sample is generally detected and quantified by quantitative real‐time PCR (qPCR) with a species‐specific marker (Doi et al. 2021). This makes eDNA analysis non‐invasive, cost‐efficient, and sensitive biomonitoring compared to traditional surveys (Yamamoto et al. 2016; Rourke et al. 2022; Hidaka et al. 2024). Nevertheless, the practical application of eDNA‐based abundance estimation is in its infancy, likely because of a limited understanding of the mechanisms producing the variability in estimated eDNA concentrations (Mathieu et al. 2020; Burian et al. 2021).

The variability arises at multiple hierarchical levels in the eDNA analysis. In addition to differences in eDNA quantifications between sites and time points caused by spatiotemporal changes in the species distribution and abundance (Shelton et al. 2019; Jo et al. 2026), eDNA concentration can also vary between replicated water samples obtained from the same site and even between PCR replicates derived from the same water sample. The inter‐sample variation is likely to be derived from the patchy distribution of eDNA particles that are composed of various particle sizes and sources, as well as the variability in filtration and extraction efficiencies after water sampling (Eichmiller et al. 2016; Furlan et al. 2016; Jo 2025). The within‐sample (inter‐PCR replicate) variation can result from the qPCR measurement error, potentially relating to the bias and uncertainty about estimating the target eDNA concentration in a sample (Mauvisseau et al. 2019; Jo et al. 2024). Such technical noises at sampling and measurement steps may mask the ’true’ difference in organism abundance between different sites and time points and weaken the link between eDNA concentration and organism abundance in the field. Therefore, understanding how sampling and PCR steps can affect the variability in eDNA quantification is essential for improving the precision of eDNA quantification and refining the quantitative performance of eDNA analysis.

Some laboratory studies compared the relative variation in eDNA quantification (coefficient of variation [CV]; standard deviation [SD] divided by the mean) between different levels (Takahashi et al. 2021; Murakami et al. 2022; Jo et al. 2024). According to them, the CV values tended to be lower at the sample level (between water samples derived from the same experimental tank) and even lower at the PCR level (between PCR replicates derived from the same sample) than at the tank level (between tank replicates). Although few in number, studies comparing variations in eDNA concentrations in natural environments across different levels have also indicated that much of the observed variation can be attributed to differences in sampling sites and time points, and that technical noise at the sampling and measurement steps is relatively small (Shelton et al. 2019; Ogonowski et al. 2023). According to these laboratory and field studies, variations in eDNA concentrations are likely to derive mainly from the spatial distribution of individuals and their dynamics and increase on a larger spatiotemporal scale.

This does not necessarily mean that variations derived from the sampling and measurement steps are negligible for experimental design. Even if their contribution seems to be small, such variation may depend on specific factors, potentially introducing systematic bias or error into observational results. For example, the CV of eDNA concentrations is known to be negatively correlated to mean values (Mauvisseau et al. 2021; Brys et al. 2023; Jo 2023; Van Driessche et al. 2023), meaning that variations in eDNA concentrations is negatively concentration‐dependent. However, it remains unclear whether and how this trend varies across hierarchical levels (sampling sites and time points, replicate samples, and PCR replicates). Understanding how variation changes with concentration helps quantify the degree of heterogeneity in eDNA distribution and identify which steps in the workflow should be prioritized to reduce observational error. Moreover, knowledge of environmental factors driving variation in eDNA concentration remains scarce, compared to that for eDNA production and persistence (e.g., Collins et al. 2018; Jo et al. 2020). Understanding how variation changes with environmental parameters can also inform the proper design of sampling and PCR strategies under different environmental conditions. Taken together, these structural characteristics of variation in eDNA concentrations provide key insights into distinguishing between systematic and stochastic sources of variability, thereby contributing to the optimization of sampling and measurement design.

The present study aimed to assess the magnitude, structure, and environmental dependence of variability in eDNA concentration in the natural environment at multiple levels. To this end, we conducted spatiotemporally replicated water sampling at the coastal ecosystems and quantified Japanese jack mackerel ( *Trachurus japonicus* ) eDNA concentrations in the seawater samples. The fish is regarded as an ecologically and economically important marine species in East Asia including Japan (Zhang and Lee 2001; Sassa and Konishi 2006) and one of the dominant species in this area, confirmed by a long‐term underwater visual census and eDNA metabarcoding (Masuda 2008; Yamamoto et al. 2017). We first conducted a variance component analysis to compare the relative contributions of each level (PCR replicates, replicated samples, and sampling sites and times) to the total variance in observed eDNA concentration. We then quantified the relationship between eDNA concentration and its variance using Taylor's Power Law (TPL) and compared the concentration dependence of the variance across levels. We also investigated environmental factors affecting the variability in eDNA concentration at the sample and PCR levels. Based on the results, we further discussed how we could improve the eDNA quantification result in the field and the interpretation of species distribution and abundance.

## 2 Materials and Methods

### 2.1 Experimental Design and Water Sampling

**FIGURE 1:**
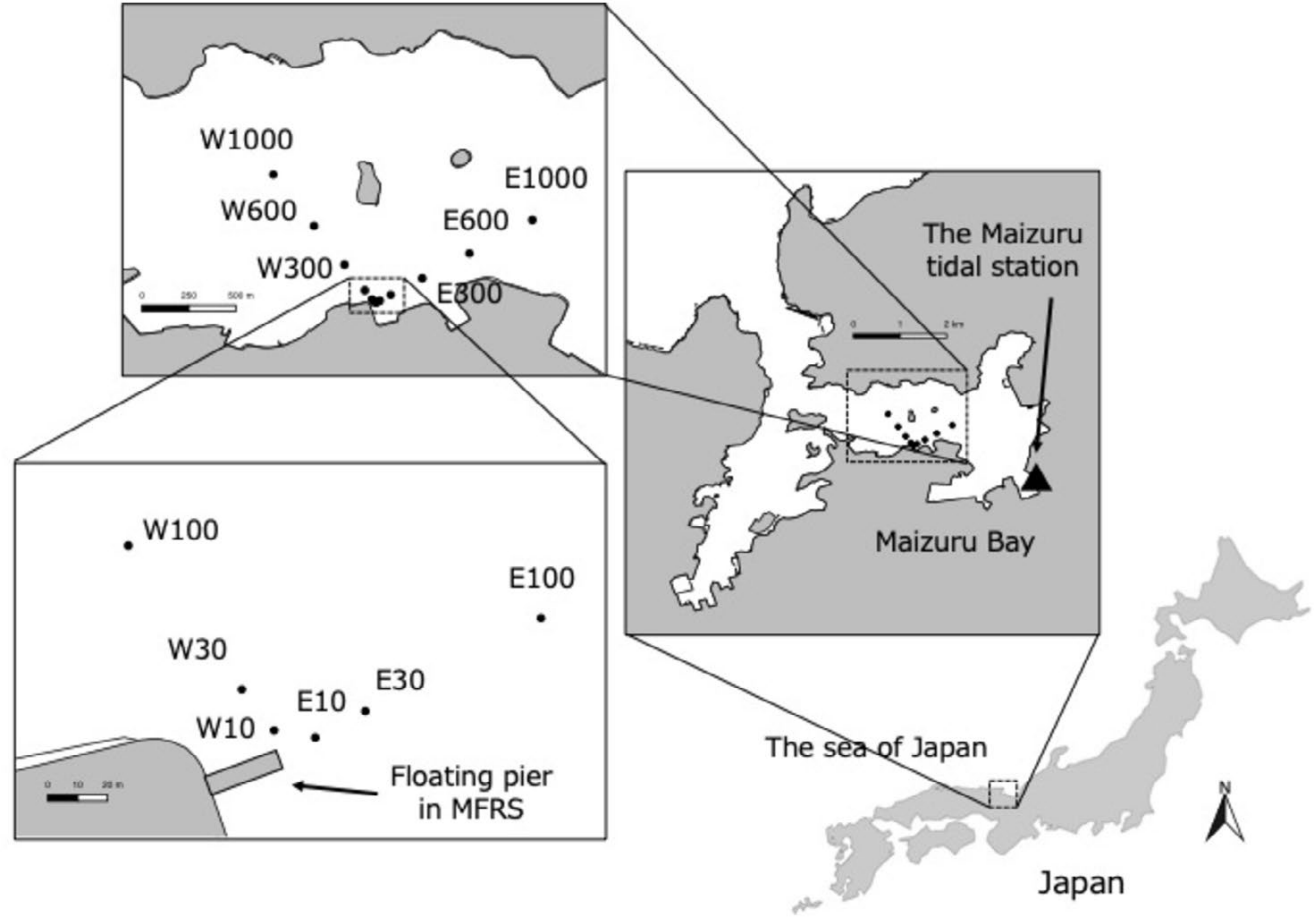
Map of the study area. Black dots indicate the seawater sampling sites. A black triangle indicates the Maizuru tidal station where the tidal level in Maizuru Bay is recorded by the Japan Meteorological Agency. The map was created by using QGIS (version 2.14.7‐ Essen) based on a dataset downloaded from the National Land Information Division (https://nlftp.mlit.go.jp/ksj/).

All seawater samples were derived from Murakami et al. (2019). The study originally examined the dispersal distance and persistence time of eDNA released from a cage housing the striped jack ( *Pseudocaranx dentex* ) in Maizuru Bay (Kyoto Prefecture, Japan). We re‐purposed the samples to quantify *T. japonicus* eDNA concentrations in this area because the species are known to be dominant in this area (Masuda 2008; Yamamoto et al. 2017). The study area is in a protected inner bay and the sea bottom is mainly composed of muddy silt. From the tip of a floating pier at the Maizuru Fisheries Research Station, Kyoto University (MFRS), which was in front of the bay, twelve sampling sites were selected at six linear distant points (10, 30, 100, 300, 600, and 1000 m from MFRS) extending northwest and northeast (six in each direction; Figure 1). On June 9, 2015, triplicate approximately 2 L of surface seawater samples were collected from each site using a ladle (3.2 L in capacity) on the boat. The seawater samples were immediately placed in a cooler box on ice to minimize the degradation of eDNA until filtration. We repeated the water sampling roughly 1, 2, 4, 8, 24, 48, 49, 50, 51, 53, 57, and 73 h after the first sampling (13‐time points in total; Table S1). The sampling time intervals stemmed from the original purpose of Murakami et al. (2019). It took 22–34 min to visit all the sites and collect water samples per sampling time point.

One liter of each seawater sample was then subjected to filtration with a 47‐mm‐diameter GF/F glass microfiber filter (nominal pore size 0.7 μm; GE Healthcare Life Science, Japan) approximately 40 min after water sampling (30 min on board and 10 min for transportation to the laboratory). At each time point and transect, 1 L of artificial seawater (MARIN MERIT, Matsuda, Japan) was also filtered as a filtration negative control. All filtered samples were immediately covered with aluminum foil and kept at −20°C until DNA extraction. Throughout the experiments, we wore disposable gloves to collect and filter water samples and bleached the filtering devices before every use in 0.1% sodium hypochlorite solution for at least 5 min, followed by a rinse with tap water and distilled water (Jo et al. 2021). Detailed information on the eDNA sampling procedure can be seen in Murakami et al. (2019).

Using a direct‐reading total water quality meter (AAQ‐RINKO(AAQ177), JFE Advantech Co. Ltd., Japan), we also measured environmental parameters (water temperature [WT; °C], salinity, conductivity [mS/cm], seawater density [kg/m^3^], chlorophyll‐α [μg/L], turbidity [FTU], pH, dissolved oxygen [DO; mg/L], and photon flux density [PFD; μmol/m^2^/s]) from the sea surface for each sampling event (12 sites × 13 time points). Since PFD is the number of light quanta passing through a unit area in unit time and is most commonly used as a measure of light intensity (Tardieu et al. 1999), the PFD is simply referred to as light intensity hereafter. Additionally, we obtained the tidal height [cm] in the study area (measured at the Maizuru tidal station; Figure 1) at each sampling time point from the web page of the Japan Meteorological Agency (Table S1; Figure S1).

### 2.2 Laboratory Analyses

Total eDNA on the filter was extracted using the DNeasy Blood and Tissue Kit (Qiagen, Germany), according to the method described by Yamamoto et al. (2016). We then estimated *T. japonicus* eDNA concentration in a water sample by quantifying the copy number of cytochrome b (CytB) genes using a LightCycler 96 System (Roche Diagnostics, Mannheim, Germany). Each 20 μL of TaqMan reaction contained 2 μL template DNA, a final 900 nM concentration of both the forward and the reverse primers, and 125 nM of TaqMan probe in 1 × Gene Expression Master Mix (Life Technologies). The primers/probe set amplified and detected a 127‐base pair fragment of the CytB genes from the target species (Jo et al. 2020). We simultaneously analyzed 2 μL of pure water as a negative PCR control, as well as a dilution series of standards containing 3 × 10^1^ to 10^4^ copies of a linearized plasmid containing synthesized artificial DNA fragments from the partial CytB gene of target species as quantification standards (Yamamoto et al. 2016). All eDNA samples, standards, and negative controls were subjected to the quantitative real‐time PCR (qPCR) in triplicates. The qPCR thermal condition was as follows: 2 min at 50°C, 10 min at 95°C, 55 cycles of 15 s at 95°C and 1 min at 60°C. Different laboratory rooms were used for water filtration, DNA extraction, and qPCR to prevent cross‐ and carryover‐contamination. Throughout the qPCR experiments, the *R* ^2^ value and amplification efficiency ranged from 0.998 ± 0.004 and 91.6% ± 3.5%, respectively (mean ± SD). We confirmed that none of the filtration and PCR negative controls were amplified.

### 2.3 Statistical Analyses

All statistical analyses were performed using R version 4.2.2 (R Core Team 2023).

#### 2.3.1 Variance Component Analysis

To partition the relative contributions of each level (PCR replicates, replicated samples, and sampling sites and times) to the total variance in observed eDNA concentration, we ran a linear mixed‐effect model (LMM) using the *lmer* function in the *lmerTest* package (Kuznetsova et al. 2017). Our dataset has a hierarchical structure; triplicate water samples were collected at 12 sites, which was repeated across 13 time points, and triplicate eDNA concentration measurements were estimated from each water sample using qPCR. In the LMM, eDNA concentration per PCR well (copies/2 μL template DNA) was set as the objective variable, and sampling site, time point, sample ID, and their crossed effects were included as the random intercept terms (Table S1). No variable was included as the fixed effect. Model formula is described as follows:

$$\text{eDNA concentration}\;\mathrm{per}\;\mathrm{PCR}\;\text{well}\sim 1+\left(1|\text{sampling site}\right)+\left(1|\text{time point}\right)+\left(1|\text{sampling site}:\text{time point}\right)+\left(1|\;\text{sampling site}:\text{time point}:\text{sample}\;\mathrm{ID}\right)$$

where residual variance refers to the variation arising from steps following water sampling and filtration (primarily between qPCR replicates).

Including our dataset, eDNA concentration data is typically characterized by over‐dispersion and zero‐inflation (Furlan et al. 2016; Song et al. 2017). With regard to the heterogeneous nature of eDNA distribution, assuming normality may lead to inaccurate modeling and inference (Bylemans et al. 2025). We therefore ran a generalized linear mixed‐effect model (GLMM) with the negative binomial (NB) error distribution to complement the LMM result. Model formula is similar to the above. As the NB‐based GLMM does not account for residual variance, we compared the relative variances in eDNA concentrations at each level other than the PCR level between the LMM and NB‐based GLMM. We also ran additional LMMs for variance component analysis of high‐ and low‐concentration subset data—divided based on the average eDNA concentration across the entire dataset (8.53 copies per reaction), taking into account the results shown in the section *Concentration dependence assessment*.

#### 2.3.2 Concentration Dependence Assessment

We then compared the concentration‐dependence of the variance (i.e., heterogeneity in eDNA concentration) across levels, using the TPL framework. The TPL was originally conceived to describe the distributional characteristics of a range of different organisms, which has been established in a variety of taxa and ecosystems (e.g., Marquet et al. 2005; Cobain et al. 2019) and has applied to the characteristics of non‐organismal particles, such as water clarity (Glines et al. 2024). When a population exhibits a random (Poisson) distribution, variability in the population density is similar among observed sites and its variance (*σ* ^2^) is proportional to the density (mean; *μ*). In contrast, when a population exhibits a patchy distribution, variability in the population density differs between the sites and its variance increases more rapidly than the density does. These distribution patterns can be expressed as a power law, with the coefficient *α* (*α* > 0) and the scaling exponent *β*, as follows:

$$\sigma^{2}=\alpha \mu^{\beta }$$

Accordingly, the log‐transformed variance is linearly related to the log‐transformed mean with the intercept *α* and the slope *β*. The scaling exponent *β* represents a relative increase in the variance to an increase in the mean. If *β* = 1, eDNA particles are considered to distribute randomly (i.e., approaching the Poisson distribution). An increasing *β* (larger than 1) represents their patchy distribution beyond random, whereas a decreasing *β* (smaller than 1) represents their uniform or more even distribution.

We calculated the mean and variance of eDNA concentrations at qPCR, sample, spatial, and temporal levels. At the qPCR level, the mean and variance were calculated based on the triplicates of qPCR measurements derived from the same seawater sample. At the sample level, the mean and variance were calculated based on the triplicates of seawater samples collected from the same site and time point, where eDNA concentration in each seawater sample was calculated by averaging the corresponding qPCR triplicates. At the spatial and temporal levels, the mean and variance were respectively calculated based on the 12 sampling sites at the same time point and the 13 sampling time points at the same site, where eDNA concentration at each site and time point was calculated by averaging the corresponding replicated samples. The standardized major axis (SMA) regressions, which account for the response and explanatory variables with equal magnitude of random variation, were then run for the mean and variance (both log‐transformed) at each of the levels using the *smatr* package (Warton et al. 2012).

From the scaling exponent (*β*) in the TPL, the relationship between the mean value (eDNA concentration) and its CV value can be derived as follows:

$$\mathrm{CV}=\frac{\sqrt{\sigma^{2}}}{\mu }\propto \mu^{\frac{\beta }{2}-1}$$

Therefore, when *β* is less than 2 (= $\frac{\beta }{2}-1$ < 0), the CV decreases as the eDNA concentration (*μ*) increases. In contrast, when *β* is equal to 2, the CV remains constant regardless of the concentration, and when *β* is greater than 2, the CV increases with the concentration. The CV—mean relationship at each level was assessed by calculating the Pearson correlation coefficient.

#### 2.3.3 Environmental Dependence Assessment

We investigated environmental factors affecting the variability in eDNA concentration (CV value) at the sample and PCR levels. The CVs at the spatial and temporal levels were not analyzed because of their limited sample sizes (*n*_CV_ = 13 and 12, respectively). At the PCR level, LMM was run using the same R function as above. The CV value (log‐transformed) was included as the objective variable and temperature, salinity, chlorophyll‐α, turbidity, DO, light intensity, and tidal height were included as the fixed effects. Turbidity and light intensity were log‐transformed given their right‐skewed distribution (Figure S1). Sampling sites and time points, as well as their crossed effect, were included as the random effects. At the sample level, a linear model with the same formula as above was run. All the environmental variables were centered to the mean value before the analysis. As high levels of correlations were observed between some of the environmental variables in the fixed effect, we removed conductivity and seawater density from the model for the PCR‐level CV and conductivity, seawater density, and chlorophyll‐α from the model for the sample‐level CV, respectively. VIFs (variance inflation factors) of the remaining variables were less than 3 for both models.

## 3 Results

### 3.1 Variance Component Analysis

Of 468 seawater samples (composed of 1404 qPCR replicates in total), 380 samples showed successful qPCR amplification of *T. japonicus* eDNA (triplicates in 177, duplicates in 136, and single replicate in 67) and 88 samples did not (Table S1). Of 156 sampling events (composed of 12 sampling sites and 13 time points), *T. japonicus* eDNA was detected in 145 events (triplicates in 108, duplicates in 19, and single replicate in 18) and was not done in 11 events. In the LMM, 93.96% of the total variance in eDNA concentration was explained by the sampling sites, time points, and their crossed effects, while the replicated water samples and qPCR replicates explained only 6.04% of the total variance (Table 1). The similar trend was observed in the NB‐based GLMM (92.04% explained by the site and time point levels and 7.96% explained by the sample level), as well as the additional LMM using high‐concentration subset data (82.07% explained by the site and time point levels and 17.93% explained by the sample and qPCR levels). In contrast, the relative contribution of the PCR level to the total variance increases greatly in the low‐concentration subset data (70.12%).

**TABLE 1:** Summary of the variance component analyses based on the LMM (a), NB‐based GLMM (b), and additional LMMs using high‐ and low‐concentration subset data (c and d).

	Groups (Name)	(a) LMM (all data)			(b) NB‐based GLMM (all data)		
		Variance	SD	Variance (%)	Variance	SD	Variance (%)
Random effects	Time point: Sampling site (Intercept)	381.19	19.52	50.01%	0.722	0.850	36.90%
	Time point (Intercept)	332.38	18.23	43.60%	0.895	0.946	45.75%
	Sampling site (Intercept)	2.66	1.63	0.35%	0.184	0.428	9.38%
	Time point: Sampling site: Sample ID (Intercept)	22.48	4.74	2.95%	0.156	0.395	7.96%
	Residual	23.55	4.85	3.09%	(Dispersion parameter: 1.36)		

	Variable	Estimate	SE	*t* value	Estimate	SE	*z* value
Fixed effect	Intercept	8.526	5.32	1.603	0.863	0.301	2.866

	Groups (Name)	(c) LMM (high‐concentration)			(d) LMM (low‐concentration)		
		Variance	SD	Variance (%)	Variance	SD	Variance (%)
Random effects	Time point: Sampling site (Intercept)	730.9	27.0	50.75%	0.769	0.877	12.49%
	Time point (Intercept)	451.2	21.2	31.33%	0.666	0.816	10.82%
	Sampling site (Intercept)	0.0	0.0	0.00%	0.201	0.449	3.27%
	Time point: Sampling site: Sample ID (Intercept)	121.7	11.0	8.45%	0.203	0.450	3.29%
	Residual	136.5	11.7	9.48%	4.314	2.077	70.12%

	Variable	Estimate	SE	*t* value	Estimate	SE	*t* value
Fixed effect	Intercept	19.0	7.1	2.672	2.227	0.280	7.952

*Note:* Residual variance is not estimated by the NB‐based GLMM owing to the model characteristics.

### 3.2 Concentration Dependence Assessment

**FIGURE 2:**
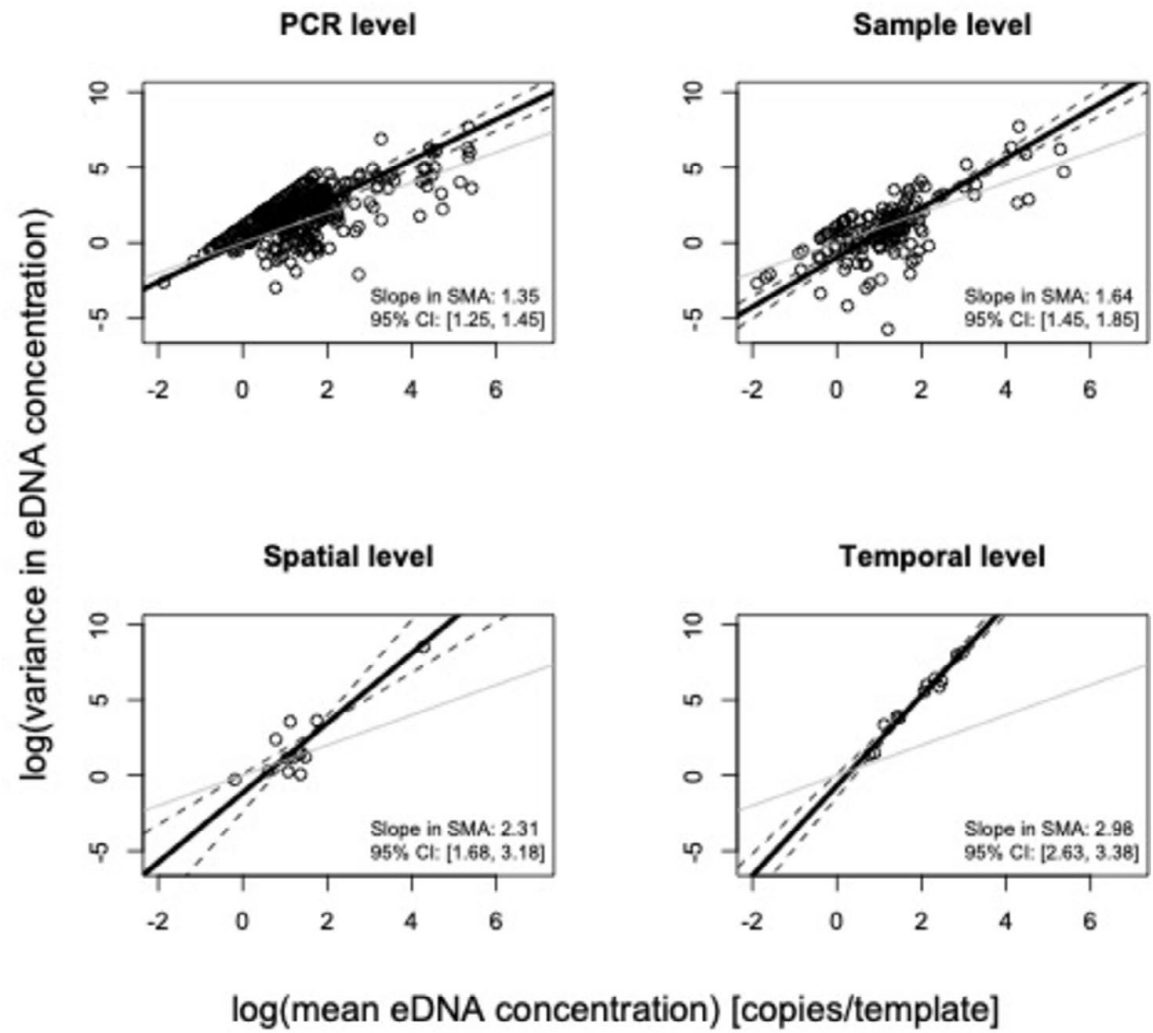
The relationships between the mean eDNA concentration and its variance (both log‐transformed) at the PCR (upper left), sample (upper right), spatial (lower left), and temporal (lower right) levels. The regression lines and their 95% CIs are shown in solid and dashed black lines. Solid gray lines indicate 1:1 line. The regression slopes correspond to *β* in the TPL framework.

**FIGURE 3:**
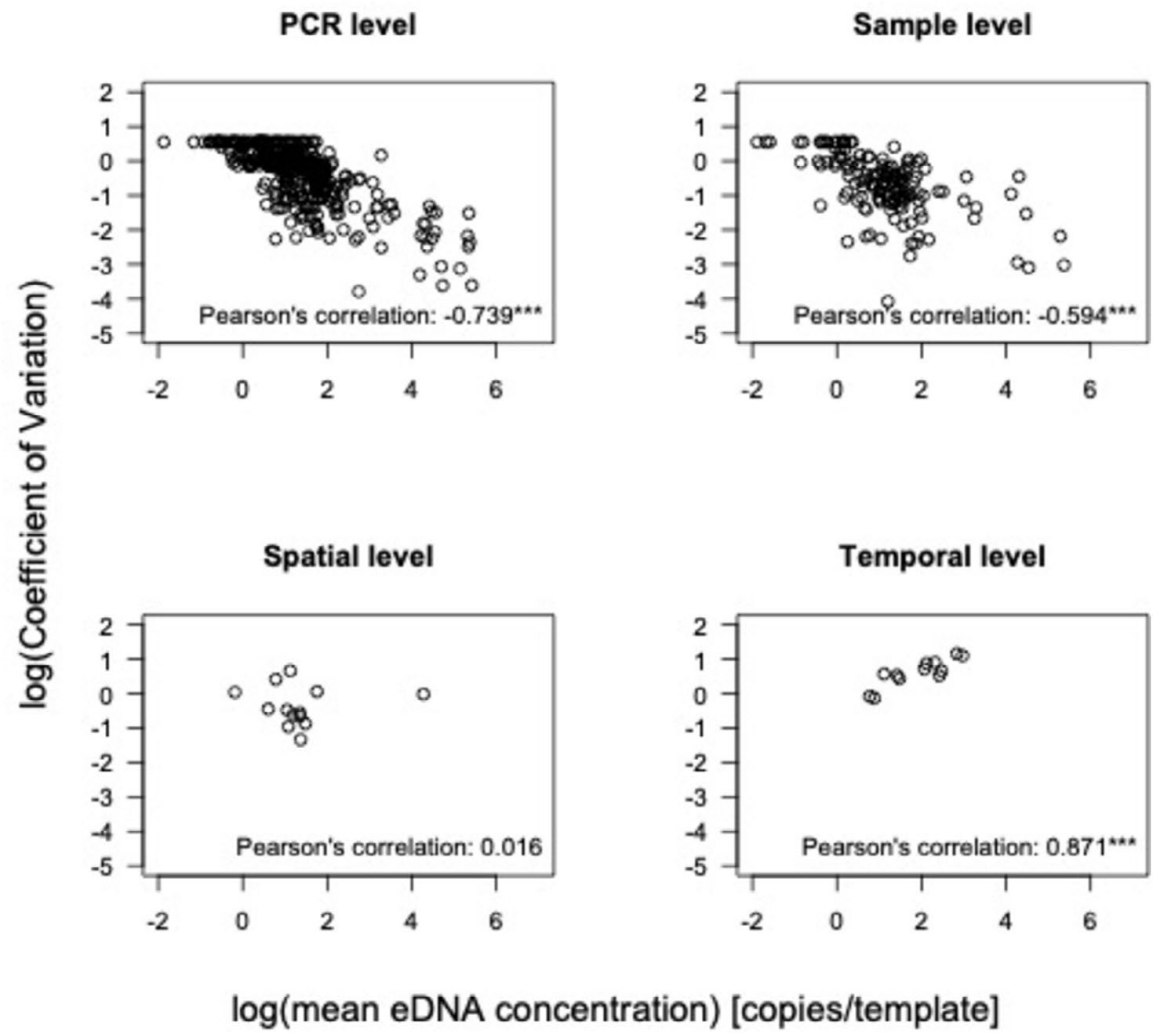
The relationships between the CV value and the mean eDNA concentration (both log‐transformed) at the PCR (upper left), sample (upper right), spatial (lower left), and temporal (lower right) levels. Pearson's correlation coefficient and its statistical significance are shown in each of the boxes.

Based on the TPL framework, the scaling exponent *β* (a relative increase in the variance to an increase in the mean eDNA concentration) was quantified. The *β* estimates were 1.35 (95% CI: [1.25, 1.45]) at the PCR level, 1.64 (95% CI: [1.45, 1.85]) at the sample level, 2.31 (95% CI: [1.68, 3.18]) at the spatial level, and 2.98 (95% CI: [2.63, 3.38]) at the temporal level (Figure 2). The regression slopes (*β*) were significantly greater than 1 at all levels and increased depending on the scale of interest. Based on the estimates, it is expected that the CV of eDNA concentration decreases in the high‐concentration range at the PCR and sample levels, remains concentration‐independent at the spatial level, and increases in the high‐concentration range at the temporal level. The expected CV–mean relationships were supported by Pearson's correlation coefficients and their statistical significances (Figure 3).

### 3.3 Environmental Dependence Assessment

**FIGURE 4:**
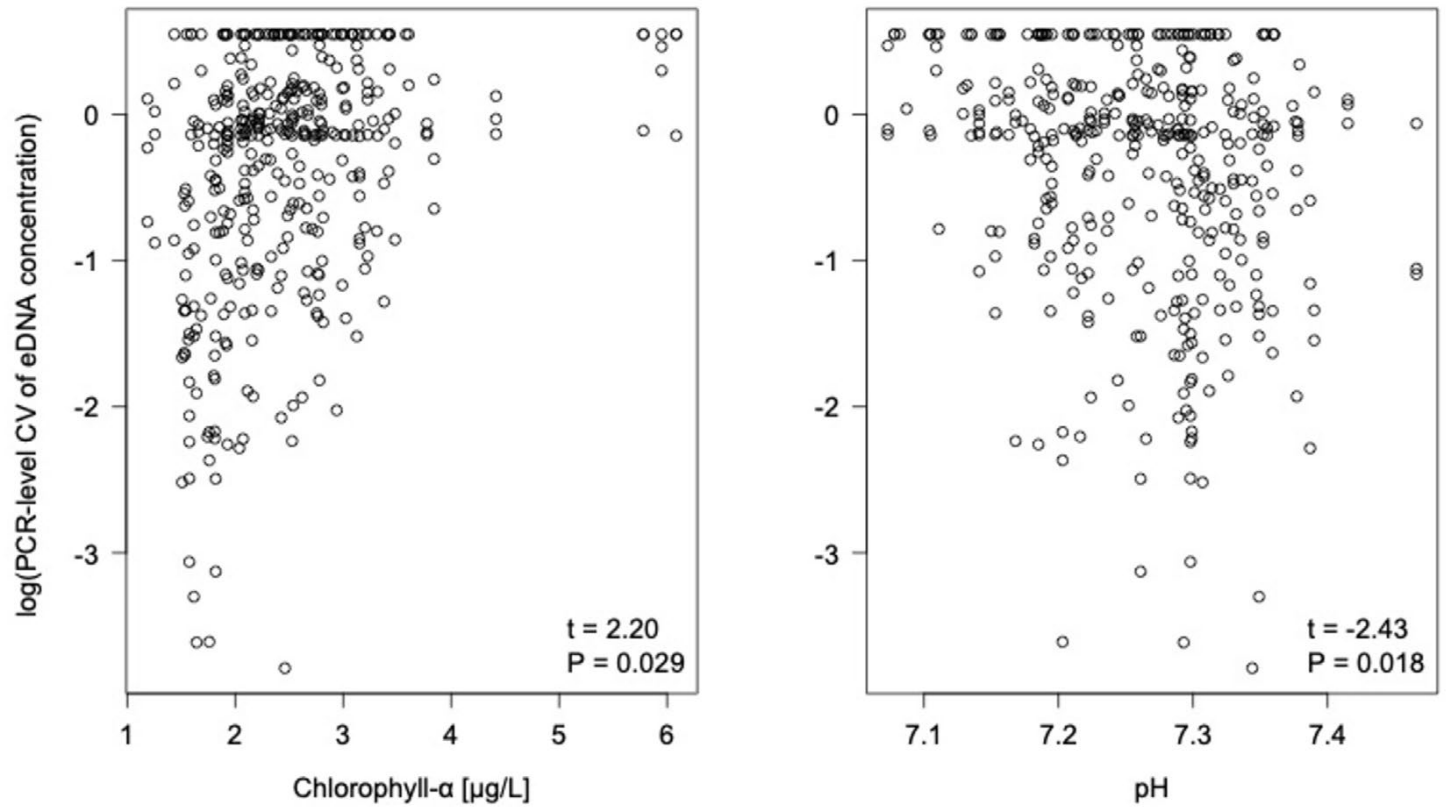
The relationships between the CV value (log‐transformed) and chlorophyll‐α (left) and pH (right) at the PCR level. The *t* values and the corresponding *p* values of the variables in the LMMs were respectively represented in each of the boxes.

The PCR‐level CV significantly increased with chlorophyll‐α (*t* = 2.20; *p* < 0.05) and negatively with pH (*t* = −2.43; *p* < 0.05) (Table 2a; Figure 4). On the other hand, no environmental variable significantly influenced the sample‐level CV (*p* > 0.1) (Table 2b). VIFs ranged from 1.24 to 2.83 in the LMM for the PCR‐level CV and 1.08 to 2.07 in the linear model for the sample‐level CV, respectively.

**TABLE 2:** Effects of environmental parameters on the CV values at the PCR (a) and sample (b) levels.

		(a) PCR‐level CV (log‐transformed)				(b) Sample‐level CV (log‐transformed)			
	Groups (Name)		Variance	SD					
Random effects	Time point: Sampling site (Intercept)		0.059	0.243					
	Time point (Intercept)		0.159	0.398					
	Sampling site (Intercept)		0.004	0.066					
	Residual		0.445	0.667					

Fixed effects	Variable	VIF	Estimate	SE	*t* value	VIF	Estimate	SE	*t* value
	(Intercept)		−0.323*	0.120	−2.701		−0.690***	0.073	−9.440
	Temperature	1.27	0.096	0.163	0.585	1.76	0.179	0.143	1.252
	Salinity	1.34	0.221	0.155	1.422	1.08	−0.247	0.165	−1.498
	Chlorophyll‐α	2.83	0.276*	0.126	2.201				
	Turbidity (log)	1.85	−0.199	0.145	−1.377	1.12	−0.299	0.194	−1.546
	pH	1.24	−1.731*	0.713	−2.429	1.34	−1.081	1.083	−0.998
	DO	1.58	−0.009	0.149	−0.061	2.07	0.092	0.233	0.395
	Light intensity (log)	1.61	−0.028	0.101	−0.281	1.96	0.111	0.098	1.142
	Tidal height	1.33	0.021	0.037	0.568	1.84	−0.007	0.027	−0.260

*Note:* Asterisks represent the statistical significance of the variables (***p < 0.001; *p < 0.05).

## 4 Discussion

### 4.1 Magnitude and Structure of eDNA Concentration Variability

When using all dataset, the sampling and qPCR processes contributed to less than 10% of the total variability in eDNA concentration. The result is not surprising, given that our target fish species are known to exhibit drastic changes for feeding and predation avoidance in their distribution throughout the day in Maizuru Bay, including the sampling sites (Jo et al. 2026), and is largely consistent with previous field surveys (Shelton et al. 2019; Ogonowski et al. 2023).

In contrast, our study pointed out that these results are not necessarily generalized and may depend on the range of observed eDNA concentrations. First, a comparison of the scaling exponent *β* across levels under the TPL framework has gained new insights into the mechanisms driving the variation in eDNA concentration. The concentration‐dependence of eDNA concentration variability differs significantly between PCR, sample, spatial, and temporal levels, and that the relative change in this variability with respect to eDNA concentration increases in proportion to the scale. Larger spatial and temporal scales integrate multiple sources of variability, including biological processes, environmental heterogeneity, and physical transport, resulting in both greater variance and stronger mean‐dependent scaling at the spatial and temporal levels. In our dataset, the heterogeneous distribution of individuals (eDNA sources) by such diel vertical and horizontal migrations, as well as advection and dilution effects (Jo 2025), should substantially affect the variation in eDNA concentration across sampling sites and time points. On the other hand, interestingly, although the contribution to the total variance in eDNA concentration was similar at both the sample and PCR levels (Table 1), the concentration‐dependence was significantly greater at the sample level than at the PCR level, as indicated by the non‐overlapping CIs (Figure 2). This indicates that, although the magnitudes are small in both levels, the structures of eDNA concentration variability differ between the sample and PCR levels.

The difference in concentration‐dependence across levels would have resulted in a considerable contribution of the qPCR level to the total variance in the low‐concentration range, as demonstrated by an additional LMM. Although formal LOD and LOQ values of the qPCR assay were not determined for the present dataset, greater number of measurements would be close to or below these analytical limits in the low‐concentration subset data. Increased stochasticity in detection and measurement errors associated with lower concentrations (Mauvisseau et al. 2021; Brys et al. 2023; Jo 2023; Van Driessche et al. 2023) may thus allow the qPCR step to be the dominant contributor to the variability in the low‐concentration range. The concentration‐dependent switching of relative contribution to the eDNA variability between levels may offer insights of the study design based on qPCR‐based eDNA quantification with a limited budget and resource. In cases where the target eDNA is expected to be present at low concentrations, such as with rare species or invasive alien species in the early stages of establishment, increasing the number of PCR replicates may be a relatively effective way of reducing quantification variability. Conversely, in cases where the target eDNA is expected to be present at high concentrations, such as when monitoring dominant species or during active seasons, priority should be given to sampling design (e.g., within‐site replication, sample pooling across sites and time points) rather than PCR design (Doi et al. 2017). In many eDNA research, it is common that triplicate qPCR measurements are generated from a single sample per site (Doi et al. 2021; Takahashi et al. 2023); however, depending on the situation, additional effort may be paid for the efficient and reliable eDNA quantification.

### 4.2 Environmental Dependence of eDNA Concentration Variability

In addition to the structural differences, we also assessed the effects of environmental factors on the variation in eDNA concentration at the PCR and sample levels. The positive effect of chlorophyll‐α on the PCR‐level CV could easily be explained by an increasing effect of PCR inhibition by eutrophication (Uchii et al. 2019; Wu et al. 2021), although this inhibition is likely to be minor and is unlikely to have a significant impact on the quantification performance in this study, considering the range of observed chlorophyll‐α concentrations (1.2–6.1 μg/L). Additionally, given the positive relationship between eDNA decay rate and chlorophyll‐α, as an indirect measure of microbial abundance (Kakuda et al. 2019; McKnight et al. 2024), poor DNA quality and integrity owing to microbial degradation may affect amplification efficiency, compromising PCR reproducibility and quantitative result consistency.

The negative effect of pH on the PCR‐level CV may also be explained by suppression of eDNA degradation under alkaline conditions (Strickler et al. 2015; Seymour et al. 2018), although it is unlikely that pH had a significant effect on the eDNA dynamics given its narrow range (7.1–7.4). Alternatively, as pH tended to be higher in coastal areas (Figure S2), it may have served as an indirect indicator of the PCR‐level CV. Collins et al. (2018) showed that, irrespective to season, eDNA degrades 1.6 times faster in the inshore environment than the offshore environment. Inshore waters are thought to be biochemically complex environments relative to offshore waters due to anthropogenic influences and inflows from land. Through microbial metabolic rates, spatially varying microbial community structures and nutrient conditions (e.g., phosphorus and carbon concentrations) may have accelerated the turnover and degradation of eDNA (Gilbert et al. 2012; Torti et al. 2015; Salter 2018).

In contrast, no environmental parameters significantly affected the sample‐level CV. Although other water quality parameters that had not been measured in our study may have influenced the sample‐level CV, the results imply that distinct processes can be involved in the eDNA concentration variability between the PCR and sample levels. Technical noise generated in the PCR step is basically random, albeit biochemical factors such as inhibitors, template DNA condition, and interactions with dissolved particles (e.g., adsorption) may introduce a degree of heterogeneity (*β*_PCR_ = 1.35 in TPL). In contrast, physical and hydrological factors, such as the fine‐scale patchiness of eDNA particles and water movement, cause variation in eDNA concentrations even within samples collected from the same site and at the same timing; this effect is likely to be greater than that at the PCR level (*β*_sample_ = 1.64 in TPL). The differences in *β* estimates in the TPL framework are broadly consistent with this inference, indicating that the variation in eDNA concentration is neither entirely random (*β* = 1) nor strongly clustered (*β* > 2), and that the degree of heterogeneity is weaker at the PCR level than at the sample level.

Environmental variables in our dataset exhibited ranges typically observed in short‐term coastal observations (Cloern et al. 2017; Carstensen and Duarte 2019); water temperature (20.7°C–23.7°C), salinity (30.5–33.2), and pH (7.1–7.5) were relatively stable, whereas turbidity (0.2–16.8 FTU) and light intensity (5.0–1459.6 μmol/m^2^/s) showed substantial spatiotemporal variation (Table S1; Figure S1). These variations were observed over a limited spatial and temporal extent (approximately 2 km and 73 h), and thus the detected relationships should be interpreted as reflecting fine‐scale variability rather than broad‐scale environmental effects. Careful consideration may also be required when interpreting the absence of significant effects, particularly for variables with relatively narrow ranges, as this may reflect limited statistical power to detect effects within such constrained gradients rather than the absence of underlying mechanisms. To our knowledge, no previous studies have directly examined environmental effects on inter‐replicate variability in eDNA concentrations. Most existing studies instead focus on eDNA decay rates under environmental gradients; for example, temperature effects have typically been evaluated across wide ranges (e.g., Strickler et al. 2015; Jo et al. 2020). However, it should be noted that while some studies have examined pH effects across wide ranges (e.g., pH 4–10; Strickler et al. 2015), others have reported significant differences even within relatively narrow pH ranges (< 1 unit; Barnes et al. 2014; McCartin et al. 2022). Furthermore, several environmental variables relevant to our study, such as light intensity (UV‐A/‐B or shade) and salinity (freshwater or seawater), have not yet been quantitatively evaluated in the context of eDNA degradation (Andruszkiewicz et al. 2017; Collins et al. 2018). Beyond the relatively calm ocean inside a semi‐closed bay targeted in this study, extending similar hierarchical experimental designs (i.e., incorporating replication at PCR, sample, spatial, and temporal levels) to ecosystems with broader environmental gradients (e.g., lakes, rivers, and open oceans) would be required for a more comprehensive evaluation of the factors driving variability in eDNA concentrations.

## 5 Conclusions and Perspectives

We assessed the magnitude, structure, and environmental dependence of the *T. japonicus* eDNA concentration variability in the coastal environment at hierarchical (PCR, sample, spatial, and/or temporal) levels. Although post‐sampling steps had a minimal influence on the total variance in the eDNA concentrations as a whole, we found that these tendencies might vary depending on the range of eDNA concentration and environmental conditions. First, differences in the levels of eDNA concentration heterogeneity based on the TPL framework suggest a concentration‐dependent survey design to minimize noise in eDNA quantification values. Given the significant variation in quantification results of low‐concentration eDNA between PCR replicates, researchers (particularly with a limited budget and resources) may need to adjust the technical steps on which their efforts should be concentrated (e.g., an increase in the number of replicates) to reduce noise in the eDNA quantification results, depending on the expected eDNA concentration range. Second, depending on the hierarchical level, variations in eDNA concentration may be caused by different factors. Whilst spatial and temporal variations in eDNA concentration could be primarily determined by ecological factors such as the distribution and dynamics of individuals, variations between samples may rely on physical and hydrological factors (transport, water movement, and fine‐scale heterogeneity), and variations between PCR replicates may rely on biochemical factors (water chemistry, compounds, and environmental gradient), respectively. Increasing the effort devoted to technical steps that are susceptible to specific environmental conditions and ecosystems may be an effective way of reducing quantification noise.

Our findings provide a practical basis for maximizing signal‐to‐noise ratios in eDNA quantification (and likely abundance estimation). While increasing PCR and sample replication has been widely recommended to improve quantification precision (Klymus et al. 2020; Yates et al. 2023), such approaches have not considered the underlying structure and environmental dependence of the variability and should not be a “one size fits all” strategy for every eDNA research. The relative importance of PCR‐ and sample‐level variability would be context‐dependent, with stochastic measurement error dominating at low concentrations and environmental heterogeneity becoming more influential at higher concentrations. These results suggest that optimal allocation of sampling effort should not be fixed, but instead tailored to expected eDNA concentration ranges and environmental conditions. By explicitly linking variability to its hierarchical structure and environmental context, our framework will contribute to establishing more efficient and adaptive sampling designs in quantitative eDNA studies for a more effective and precise monitoring of ecosystems and fisheries resources.

## Author Contributions

**Toshiaki S. Jo:** conceptualization (lead), data curation (lead), formal analysis (lead), methodology (lead), validation (lead), visualization (lead), writing – original draft (lead), writing – review and editing (equal). **Hiroaki Murakami:** data curation (supporting), funding acquisition (lead), investigation (lead), resources (lead), writing – review and editing (equal).

## Funding

This work was supported by the Sasakawa Scientific Research Grant from The Japan Science Society (Grant Number: 28‐752), JST CREST (Grant Number: JPMJCR13A2), JSPS KAKENHI (Grant Number: JP21K14899), and JST COI‐NEXT (Grant Number: JPMJPF2110).

## Conflicts of Interest

The authors declare no conflicts of interest.

## Supporting information


**Figure S1:** Histograms of environmental parameters measured in this study (water temperature [°C], salinity [‰], conductivity [mS/cm], seawater density [kg/m^3^], chlorophyll‐α [μg/L], turbidity [FTU], pH, dissolved oxygen [mg/L], light intensity [μmol/m^2^/s], tidal height [cm], from top left to bottom right).
**Figure S2:** The relationship between pH and distance from the coast. Exactly, the distance refers to the distance from the fish cage (see the manuscript and Murakami et al. (2019)), but it largely reflects the distance from the coast (the distance is greater for samples taken further offshore). LMM was run between pH values and the distance, in which sampling direction (northwest and northeast) and time points were included as the random effects. The *t* value and the corresponding *p* value of the distance effect in the LMM were represented in the box. Different colors indicate the sampling direction (light gray: northwest, dark gray: northeast).

**Table S1:** Raw qPCR data and abiotic parameters measured in this study.

## Data Availability

Raw qPCR data and environmental parameters are included in the supporting information S1.
