# Supplementary material for: Variability in Fish Environmental DNA Concentration in Coastal Ecosystems at Hierarchical Levels: Focusing on the Magnitude, Structure, and Environmental Dependence
Source: Ecol Evol. 2026 Jul 12;16(7):e74002. doi: 10.1002/ece3.74002 (PMC13357702; doi:10.1002/ece3.74002)
Supplement: Supplementary file 1 — Figure S1: Histograms of environmental parameters measured in this study (water temperature [°C], salinity [‰], conductivity [mS/cm], seawater density [kg/m3], chlorophyll‐α [μg/L], turbidity [FTU], pH, dissolved oxygen [mg/L], light intensity [μmol/m2/s], tidal height [cm], from top left to bottom right). Figure S2: The relationship between pH and distance from the coast. Exactly, the distance refers to the distance from the fish cage (see the manuscript and Murakami et al. (2019)), but it largely reflects the distance from the coast (the distance is greater for samples taken further offshore). LMM was run between pH values and the distance, in which sampling direction (northwest and northeast) and time points were included as the random effects. The t value and the corresponding p value of the distance effect in the LMM were represented in the box. Different colors indicate the sampling direction (light gray: northwest, dark gray: northeast). [file ECE3-16-e74002-s001.docx]

**Figure legends**

Figure S1. Histograms of environmental parameters measured in this study (water temperature [°C], salinity [‰], conductivity [mS/cm], seawater density [kg/m^3^], chlorophyll-α [µg/L], turbidity [FTU], pH, dissolved oxygen [mg/L], light intensity [μmol/m^2^/s], tidal height [cm], from top left to bottom right).

Figure S2. The relationship between pH and distance from the coast. Exactly, the distance refers to the distance from the fish cage (see the manuscript and Murakami et al. (2019)), but it largely reflects the distance from the coast (the distance is greater for samples taken further offshore). LMM was run between pH values and the distance, in which sampling direction (northwest and northeast) and time points were included as the random effects. The *t* value and the corresponding *P* value of the distance effect in the LMM were represented in the box. Different colors indicate the sampling direction (light gray: northwest, dark gray: northeast).

Figure S1.

Figure S2.
